# First Report of Tripled Retromolar Foramina

**DOI:** 10.7759/cureus.1440

**Published:** 2017-07-07

**Authors:** Puhan He, Joe Iwanaga, Mindy K Truong, Nimer Adeeb, R. Shane Tubbs, Koh-ichi Yamaki

**Affiliations:** 1 Harvard School of Dental Medicine, Harvard University; 2 Seattle Science Foundation; 3 Neurosurgery, Louisiana State University for medical sciences, shreveport; 4 Neurosurgery, Seattle Science Foundation; 5 Department of Anatomy, Kurume University School of Medicine

**Keywords:** mandible, canal, foramen, dental implant, oral surgery, anatomy, anatomic variation

## Abstract

The retromolar foramen (RMF) is the bony entrance of the retromolar canal, through which a neurovascular bundle runs. It is essential to locate such anatomic variants in a patient in order to avoid complications in surgery, implant placement, or anesthesia of the area. To our knowledge, there has only been one case report of supernumerary RMF, which reported one bilateral doubled retromolar foramina and one doubled left RMF. We present an extremely rare case in which a right triple RMF was observed on cone beam computed tomography in a cadaver. Diameters of the RMF were 0.8 mm, 1.0 mm and 1.1 mm, respectively. Distances from the distal edge of the third molar were measured as 4.0 mm, 3.6 mm, and 0.5 mm, respectively. RMF was not found on the left side of the mandible.

## Introduction

A variety of accessory foramina of the mandible have been reported, such as accessory mental foramina [1], lingual foramina [2], and retromolar foramina. The retromolar foramen (RMF) is the aperture of the retromolar canal (RMC) in the mandible. It is located distal to the last molar tooth in the retromolar trigone. The significance of the RMC lies in the neurovascular bundle which runs through it [3]. The RMC is a type 1 bifidity of the mandibular canal which runs through the RMC. A bifid mandibular canal is an anatomical variation in which the mandibular canal divides into two separate parts, wherein each part may contain its own neurovascular bundle [4].

After exiting from the body of the mandible, the nerve of the RMC distributes mainly to the temporalis tendon, buccinator muscle, the most posterior zone of the alveolar process, and the mandibular third molar [5]. Thus, the contents of the RMC provide innervation and vascular supply for the third molar and mucosa of the retromolar area [6]. Injury to the neurovascular bundle of the RMC during surgical procedures, such as third molar tooth extraction, implant placement, or split sagittal osteotomy, may lead to paresthesia, excessive bleeding, or traumatic neuroma. The presence of RMC may also lead to insufficient anesthesia of the mandibular teeth [7]. It is beneficial to locate the RMF and RMC prior to surgery in this area, or in order to achieve full anesthesia.

There have been very few reports of multiple RMF unilaterally in the mandible. We present here a case of tripled RMF on the right side of the mandible in a cadaver.

## Case presentation

**Figure 1:**
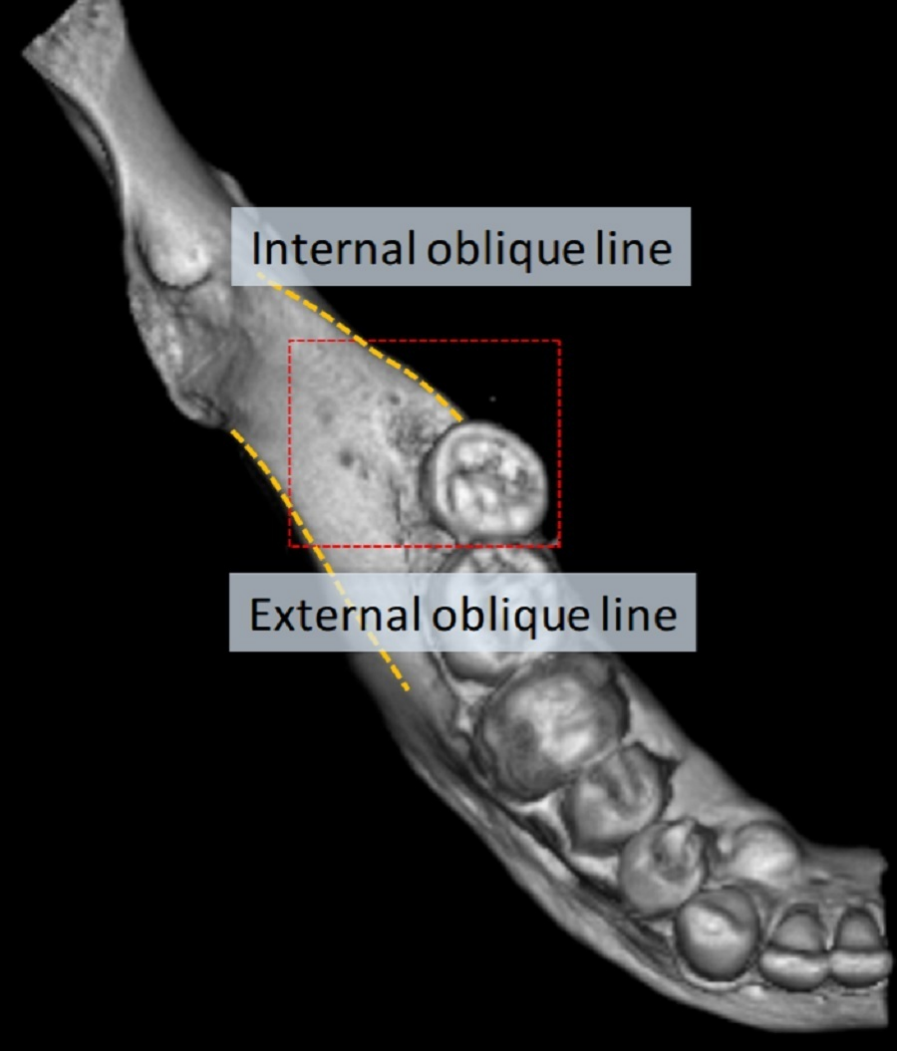
A cone beam computed tomography scan of the right mandible demonstrating triple retromolar foramina posterior to the third molar.

A mandible resected from an 84-year-old female cadaver who died from gallbladder cancer underwent cone-beam computed tomography. The scan was set at 85 kV and 6 mA. The axial images were transmitted in the digital imaging and communication in medicine (DICOM) format, and 2D images in the mandible were then reconstructed using the DICOM viewer [8]. A unique anatomical variation of triple RMF was observed at the retromolar region between internal and external oblique line on the right mandible (Figure 1).

**Figure 2:**
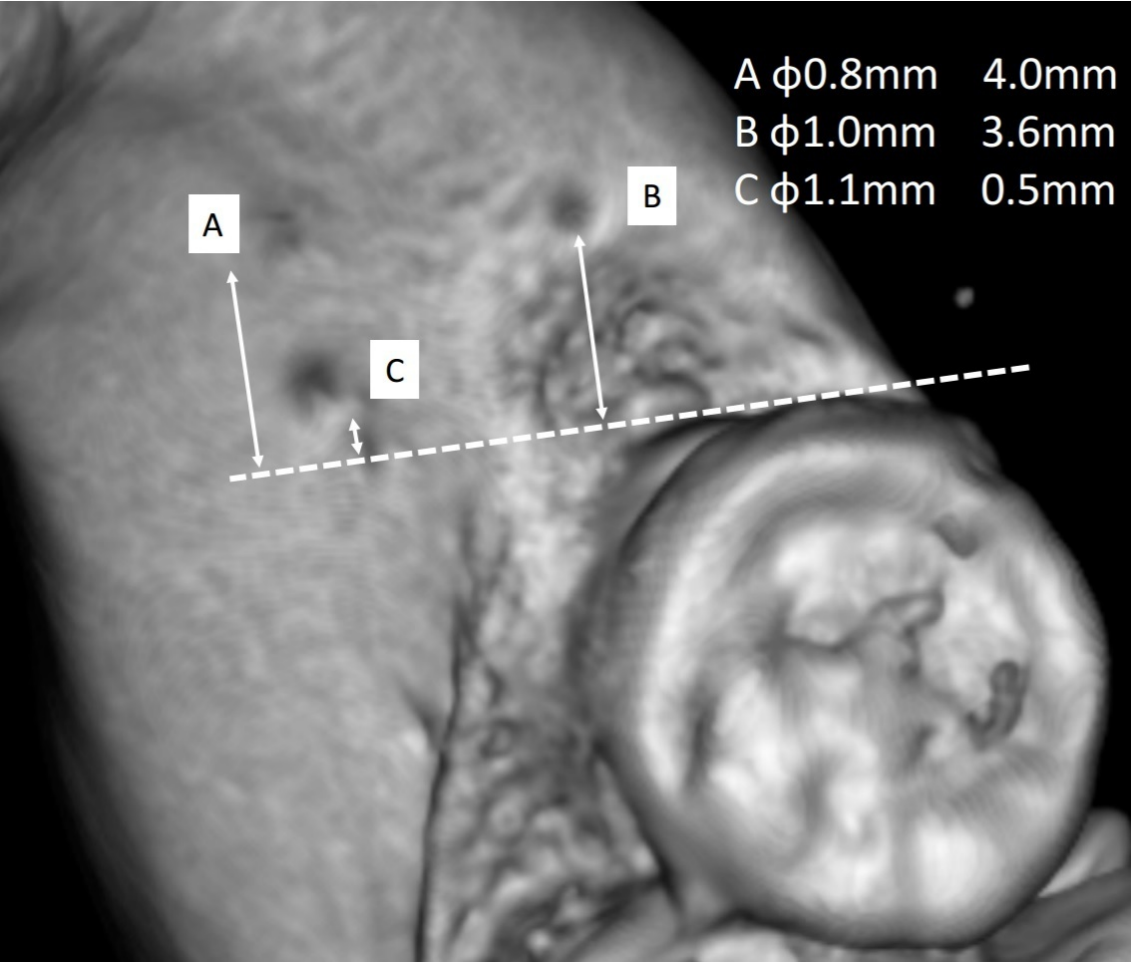
The three right retromolar foramina labeled A, B, and C were measured for 1) diameter of the foramen and 2) distance from the third molar. A) 0.8 mm, 4.0 mm, B) 1.0 mm, 3.6 mm, C) 1.1 mm, 0.5 mm.

The RMF were labeled A, B and C with diameters of 0.8 mm, 1.0 mm and 1.1 mm, respectively as shown in Figure 2. Distances from the last molar were determined by extending a horizontal line from the distal edge of the third molar. Their respective distances were measured as 4.0 mm, 3.6 mm, and 0.5 mm (Figure 2).

RMF was not found on the left side of the mandible.

## Discussion

The RMF is an important anatomic structure representing a variation in the branching of the mandibular canal. There exists a large variation in size, location, and frequency of the RMF in reports in the literature. To our knowledge, this is the only case of tripled right RMF reported in the extant literature. The first case of a bilateral doubled RMF as well as the first case of a doubled left RMF were described by Alves and Deana [3].

The frequency of the RMF described in human dry mandible studies is between 3.2% [9] and 72% [5]. The diameter of the RMF in the specimen reported herein falls within the lower limit of the range described in the literature of between 0.2 mm [10] and 3.29 mm [4]. Consistent with the findings of Haas [7], the RMF with a diameter greater than 1 mm was in a more anterior position. The specimen presented with a foramen with a diameter of 1.1 mm, which was located in a more anterior position compared to the other two foramina. The distances from the distal edge of the third molar to the foramen were less than that described by Bilecenoglu and Tuncer (4.23 mm) [6] and Schejtman, et al. (10.5 mm) [5].

## Conclusions

While there have been no reports of excessive complication due to injuring the RMF, the presence of the RMF may be implicated particularly in difficulty of anesthetization. The RMF may contain nerves that innervate the temporalis or its tendon, buccinator muscle, posterior portion of the mandible, third molar teeth, gingiva of the mandibular molars and premolars, and mucosa of the retromolar pad. This extensive network may result in failure of the standard inferior alveolar nerve (IAN) block.

The unique anatomic variation reported in this case highlights the necessity to evaluate patients for the presence of RMF and canals for unexplained mandibular pains or paresthesia. An improved anatomic understanding of the various accessory foramina along with their various locations is essential prior to performing anesthesia and surgery in the oral cavity.
